# Acetylshikonin suppresses diffuse large B-Cell Lymphoma cell growth by targeting the T-lymphokine-activated killer cell-originated protein kinase signalling pathway

**DOI:** 10.1080/21655979.2022.2034584

**Published:** 2022-02-09

**Authors:** Jieke Cui, Rong Guo, Yingjun Wang, Yue Song, Xuewen Song, Hongwen Li, Xiaoqin Song, Jiwei Li

**Affiliations:** a Department of Hematology, The First Affiliated Hospital of Zhengzhou University, Zhengzhou, China; b Department of Oncology, The First Affiliated Hospital of Zhengzhou University, Zhengzhou, China; c Department of Hematology, Affiliated Cancer Hospital of Zhengzhou University, Zhengzhou, China; d Depatment of Physical Center, The First Affiliated Hospital of Zhengzhou University, Zhengzhou, China

**Keywords:** DLBCL, TOPK, ERK, acetylshikonin, cell growth

## Abstract

Diffuse large B-cell lymphoma (DLBCL) is one of the most common causes of cancer death worldwide, and responds poorly to the existing treatments. Thus, identifying novel therapeutic targets of DLBCL is urgently needed. In this study, we found that T-lymphokine-activated killer cell-originated protein kinase (TOPK) was highly expressed in DLBCL cells and tissues. Data from the GEPIA database also indicated that TOPK was highly expressed in DLBCL tissues. The high expression levels of proteins were identified via Western blots and immunohistochemistry (IHC). TOPK knockdown inhibited cell growth and induced apoptosis of DLBCL cells with 3-(4,5-dimethylthiazol-2-yl)-5-(3-carboxymethoxyphenyl)-2-(4-sulfophenyl)-2 H-tetrazolium (MTS) and flow cytometry. Further experiments demonstrated that acetylshikonin, a compound that targeted TOPK, could attenuate cell growth and aggravate cell apoptosis through TOPK/extracellular signal-regulated kinase (ERK)-1/2 signaling, as shown by MTS, flow cytometry and Western blots. In addition, we demonstrated that TOPK modulated the effect of acetylshikonin on cell proliferation and apoptosis in U2932 and OCI-LY8 cells using MTS, flow cytometry and Western blots. Taken together, the present study suggests that acetylshikonin suppresses the growth of DLBCL cells by attenuating TOPK signaling, and the targeted inhibition of TOPK by acetylshikonin may be a promising approach for the treatment of DLBCL.

## GRAPHICAL ABSTRACT

**Figure:**
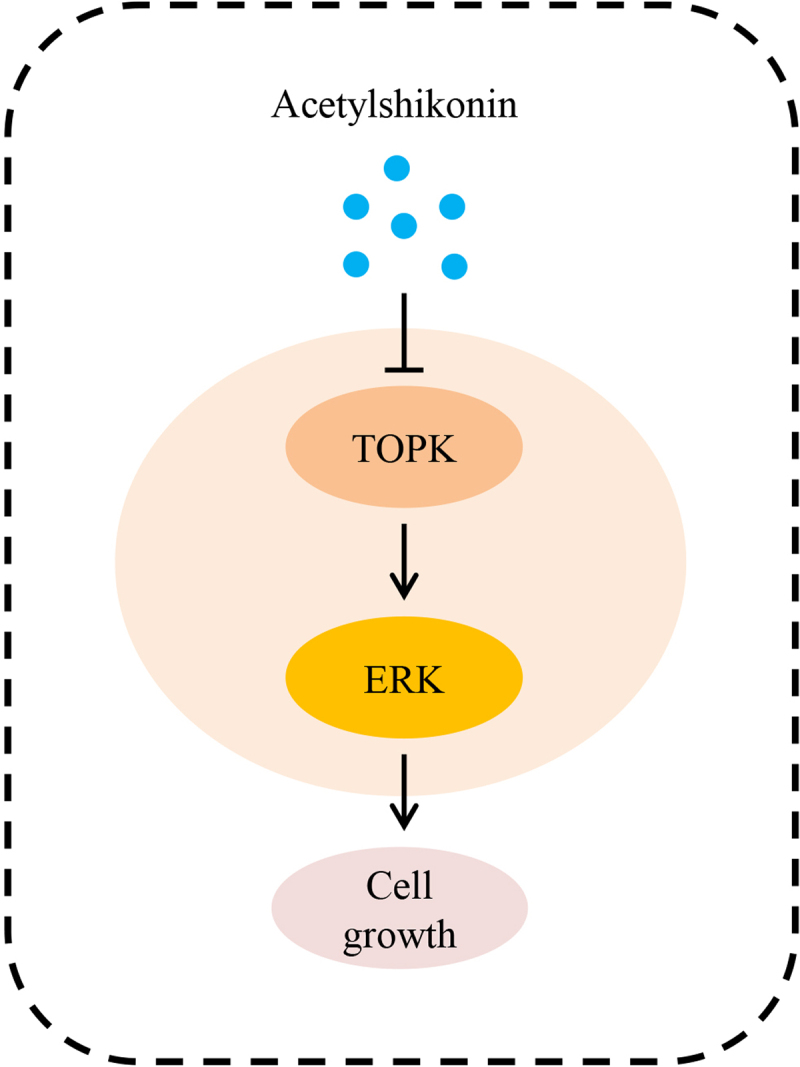

## Introduction

Diffuse large B cell lymphoma (DLBCL) is the most common type of pathology in non-Hodgkin lymphoma (NHL), representing approximately 30% of all diagnosed NHL cases. Adding rituximab to cyclophosphamide, doxorubicin, vincristine, and prednisone (R-CHOP) chemotherapy has become the standard treatment and has significantly improved the survival rate of DLBCL patients [1–3]. However, approximately 40% of patients still experience treatment failure, because of biological heterogeneity [4,5]. Thus, investigating novel biomarkers are important for the treatment of DLBCL patients.

Lymphokine-activated killer T (T-LAK)-cell-originated protein kinase (TOPK) is a member of the serine-threonine kinases of the mitogen-activated protein kinase kinase (MAPKK) family and is highly expressed in many cancers, including adult T-cell leukaemia/lymphoma (ATLL) and B cell lymphoma [6–10]. TOPK promotes cancer cell proliferation by phosphorylating ERKs [11,12]. The knockdown of TOPK activates cysteine proteases (caspase −3 and caspase −7) and finally kills cancer cells [13,14]. In primary central nervous system lymphoma (PCNSL), TOPK can be a potent novel biomarker, and its overexpression is associated with poor prognosis of PCNSL [15]. However, the underlying role of TOPK in DLBCL remains unclear.

Studies indicate that the inhibition of TOPK could be a strategy for cancer chemoprevention and treatment [16], and examine the function of TOPK inhibitors, such as OTS964, which could inhibit glioma stem cell survival, but the surviving glioma stem cells eventually start to regrow [17]. The inhibitor 3-DSC is isolated from the *Caesalpinia sappan* L. plants [18], and binds to the ATP binding pocket of TOPK; this molecule could inhibit colon cancer growth by targeting the TOPK signaling pathway *in vitro* [14]. Moreover, acetylshikonin, another TOPK inhibitor, reduces colorectal cancer cell proliferation and decreases the volume of patient-derived xenograft (PDX) tumors in mice [13]. These studies suggest that TOPK may be a promising target for anticancer therapy.

However, the expression and role of TOPK in DLBCL remains unclear. Thus, in this study, we aim to investigate the function of TOPK and acetylshikonin suppresses DLBCL cell growth by targeting the TOPK signaling pathway. We reported the crucial role of TOPK in DLBCL tumorigenesis. TOPK knockdown attenuated the malignant phenotypes of DLBCL cells, including cell proliferation and apoptotic induction. Furthermore, we demonstrated that acetylshikonin inhibited cell proliferation and induced cell apoptosis by targeting the TOPK signaling pathway. These results suggest that TOPK may be a promising molecular target in DLBCL treatment.

## Materials and Methods

### Reagents

The compound 3-DSC (cat: JOT-10796) was purchased from Chengdu Pufei De Biotech Co., Ltd (Chengdu, China), acetylshikonin (cat: YRY134) was purchased from Chengdu Yirui Biotech Co., Ltd. (Chengdu, China), and antibodies to detect total TOPK (cat: 4942, lot: 3), phosphorylated TOPK (cat: 4941, lot: 4), total ERKs (cat: 9102, lot: 4), phosphorylated ERKs (cat: 4370, lot: 2), total RSK (cat: 5528, lot: 5), phosphorylated RSK (cat: 11,989, lot: 2), total c-Jun (cat: 9165. lot: 3), phosphorylated c-Jun (cat: 3270, lot: 2), caspase-3 (cat: 9662, lot: 8), caspase-7 (cat: 12,827, lot: 12), cleaved caspase-3 (cat: 9664, lot: 4), cleaved caspase-7 (cat: 9491, lot: 4) and actin (cat: 3700, lot: 6) were purchased from Cell Signaling Technology (Beverly, MA, USA).

### Cell culture

Human DLBCL cell lines (U2932, SUDHL-6 and OCI-Ly8) and human B lymphoblast cells (WIL2S) were purchased from the Type Culture Collection of the Chinese Academy of Sciences (Shanghai, China). The cells were cultured in RPMI 1640 medium with 10% fetal bovine serum (FBS). WIL2S cells were maintained in Iscove’s modified medium with 10% human serum (NABI Biopharmaceuticals, Boca Raton, FL, USA) and 2 mM L-glutamine (Invitrogen, Carlsbad, CA, USA). All cells were cultured with a penicillin/streptomycin antibiotic mixture, penicillin (100 U ml^−1^), and streptomycin (100 μg ml^−1^) at 37°C in 5% CO_2_.

### Isolation of peripheral blood mononuclear cells (PBMCs)

Human blood (10 ml) was collected, and added 10 ml of PBS was added. PBMCs were collected by density gravity centrifugation (centrifuged at 500 × g for 30 min at room temperature.) using Ficoll-Paque (cat. no. LTS10770125, TBD science, Tianjin, China). Then, the PBMCs were washed with PBS [19].

### Immunohistochemical (IHC) staining

A DLBCL tissue array (cat: LY800b) containing 40 DLBCL samples and 20 control tissue samples (10 lymph nodes, 5 spleens, 5 tonsils; the median age was 55 years old) was purchased from Alenabio Biotechnology Co., Ltd., Tissue specimens were fixed in 10% (v/v) formaldehyde in phosphate-buffered saline, embedded in paraffin and cut into 5 μm sections. The sections were deparaffinized in xylene solution and rehydrated using gradient ethanol concentrations. Antigen retrieval was performed using sodium citrate, and the slides were then incubated with H_2_O_2_ to block endogenous peroxidases. Thereafter, TOPK (1:200) antibody was incubated for 12–16 h. After PBS washing, the sections were incubated with a horseradish peroxidase-conjugated secondary antibody (Rabbit HRP EnVision TM+, Dako, Denmark) for 30 min. After the sections were washed with PBS, they were incubated with 3,3-diaminobenzidine (DAB) substrates for 1 min and counterstained with hematoxylin to show the nuclei. After developing, all sections were observed by microscopy, and quantitative analysis was performed using the Image-Pro Premier software (v.9.0) program [13].

### Western blot analysis

Cell pellets were incubated on ice for 30 min in NP-40 cell lysis buffer and centrifuged at 12,000 × g for 10 min, and the supernatant fractions were harvested as the total cellular protein extracts. A BCA quantification kit (Solarbio, Beijing, China, cat: PC0020) was used to detect the protein concentration. The protein samples were separated by SDS-PAGE, then, transferred to polyvinylidene difluoride (PVDF) membranes (Bio-Rad, Hercules, CA, USA). The membranes were blocked with defatted milk, incubating with primary antibodies (1:1000), including TOPK, pTOPK, ERK, pERK, RSK, pRSK, c-Jun, pc-Jun, caspase 3, caspase 7, cleaved caspase 3, cleaved caspase 7 and actin antibodies, and then incubated with secondary antibodies. Images were captured with a Tanon-5200 system [20].

### Lentiviral vector construction

The TOPK (accession number NM_018492.4) sequence was synthesized by Shanghai GenePharma Co., and cloned into the pLVX vector. shTOPK.1 (ATTAGTGCATACAGAGAAGAGTT) and shTOPK.2 (GTCTGTGTCTTGCTATGGAAT) were synthesized by Shanghai GenePharma Co., Ltd., to deplete the expression of TOPK in DLBCL cells. A scrambled shRNA was used as a negative control. shRNA oligos were cloned into the pLKO vector. Then, recombinant lentivirus was generated by cotransfecting shRNA plasmids or overexpression plasmids and pHelper plasmids into HEK293T cells using lipofectamine 2000 (Invitrogen) according to the manufacturer’s instructions [21].

### Transfection

Cells were seeded in six-well plates and infected with the constructed lentivirus at a multiplicity of infection (MOI = 100). The final concentration of polybrene was 5 μg/ml. Puromycin (2 μg/ml) was added 24 h after infection, and then, stably transfected cell lines were screened. The knockdown or overexpression efficiency was determined by protein levels at 48 h after infection [21].

### Cell proliferation assay

Cells (5 × 10^3^ cells/well) were seeded in 96-well plates and treated with different concentrations (5 µM, 10 µM, 20 µM, 40 µM, 80 µM) of acetylshikonin or 3-DSC. Cell proliferation was detected at various times (24, 48, and 72 h). For each well, 20 µl of the MTS solution (Promega, Madison, WI) was added. After 1 h of incubation, 25 µl of 10% SDS solution was added, and the absorbance was detected at 492 and 690 nm with a microplate spectrophotometer [22].

### Detection of apoptosis

Flow cytometry was performed to observe cell apoptosis with an annexin V-FITC apoptosis detection kit (Beyotime, C1062S). Cells were collected and washed with cold PBS. FITC annexin V and PI were added and incubated for 15 min at room temperature in the dark. Then, the cells were analyzed by flow cytometry within 1 h. The percentages of early (annexin V-FITC positive, PI negative) and late (annexin V-FITC positive, PI positive) apoptotic cells were determined by quadrant analysis of the annexin V-FITC/PI plots using FlowJo software (Tree Star Inc, Ashland, OR) following the manufacturer’s recommended protocol. Annexin V-FITC-negative and PI-negative cells were viable, and annexin V-FITC-negative and PI-positive cells were dead [23].

### Mouse xenograft model

Twelve female BALB/c nude mice (4–6 weeks old) were obtained from Beijing Huafukang Biotechnology Co., Ltd (Beijing, China). U2932 cells (5 × 10^6^ cells/ml) were suspended in 100 μl of PBS and injected in the left flank of mice. One week after implantation, mice were randomly divided into two groups. Acetylshikonin (120 mg.kg^−1^) or vehicle was administered by gavage once a day. Tumor volume was calculated every 3 days. Tumor volumes were calculated using the formula: (length × width^2^) × 0.5. Mice were euthanized at 1 month and the tumor tissues were isolated and weighted [24].

### Statistical analysis

The results are presented as the mean ± standard deviation (SD) of 3 independent experiments, and each dosage or treatment was tested in triplicate. Statistical analyses were performed with GraphPad Prism 6. Student’s *t* test was used to analyze the significant differences, which were defined when the *p* value was less than 0.05.

## Results

### TOPK is expressed at high levels in DLBCL

**Figure 1.**
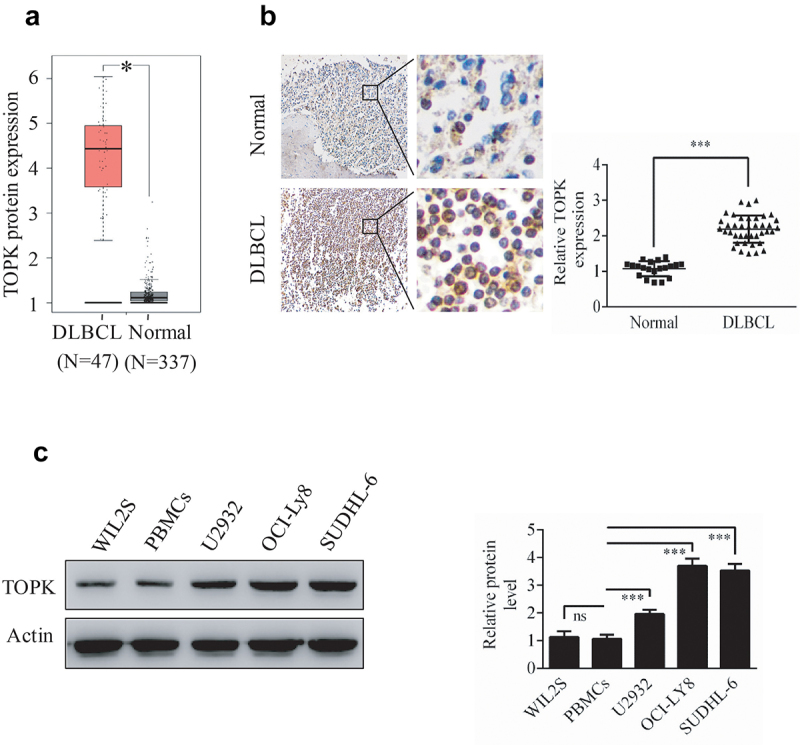
**TOPK is highly expressed in human DLBCL**. (a) The GEPIA database demonstrated the overexpression of TOPK in DLBCL tumor tissues compared to normal tissues (*p < 0.05). (b) IHC staining revealed nuclear staining of TOPK in the DLBCL tissue array, control group (20 samples), and DLBCL group (40 samples). The values were normalized to control tissue expression, and the values are represented as the mean ± S.D. (***p < 0.001). (c) TOPK protein levels were measured in DLBCL cell lines (U2932, OCI-LY8, SUDHL-6), WIL2S cell lines and PBMCs using Western blots. The data represent the mean ± S.D. for three individual experiments (ns: no significant difference, **p < 0.01, ***p < 0.001, n = 3).

TOPK is highly expressed in many kinds of cancers and shows as an emerging target for cancer-specific therapeutics [7], however, the expression and role of TOPK in DLBCL remains unclear. In this study, we aim to investigate the function of TOPK in DLBCL. To explore the role of TOPK in DLBCL, we analyzed TOPK expression in DLBCL cancer specimens and normal controls in the GEPIA dataset. As shown in Figure 1(a), TOPK expression was obviously upregulated in DLBCL cancer specimens compared with normal samples. This result was further confirmed by testing TOPK expression using IHC of the DLBCL tumor microarray that included 40 DLBCL tissues and 20 control tissues (Figure 1(b)). Similarly, TOPK expression was upregulated in DLBCL cell lines (U2932, OCI-LY8, SUDHL-6) compared with WIL2S cell lines and PBMCs at the protein level, as shown by Western blots (Figure 1(c)). These results suggest that the upregulation of TOPK expression may promote DLBCL progression.

### Silencing TOPK reduces the tumorigenic properties of DLBCL

**Figure 2.**
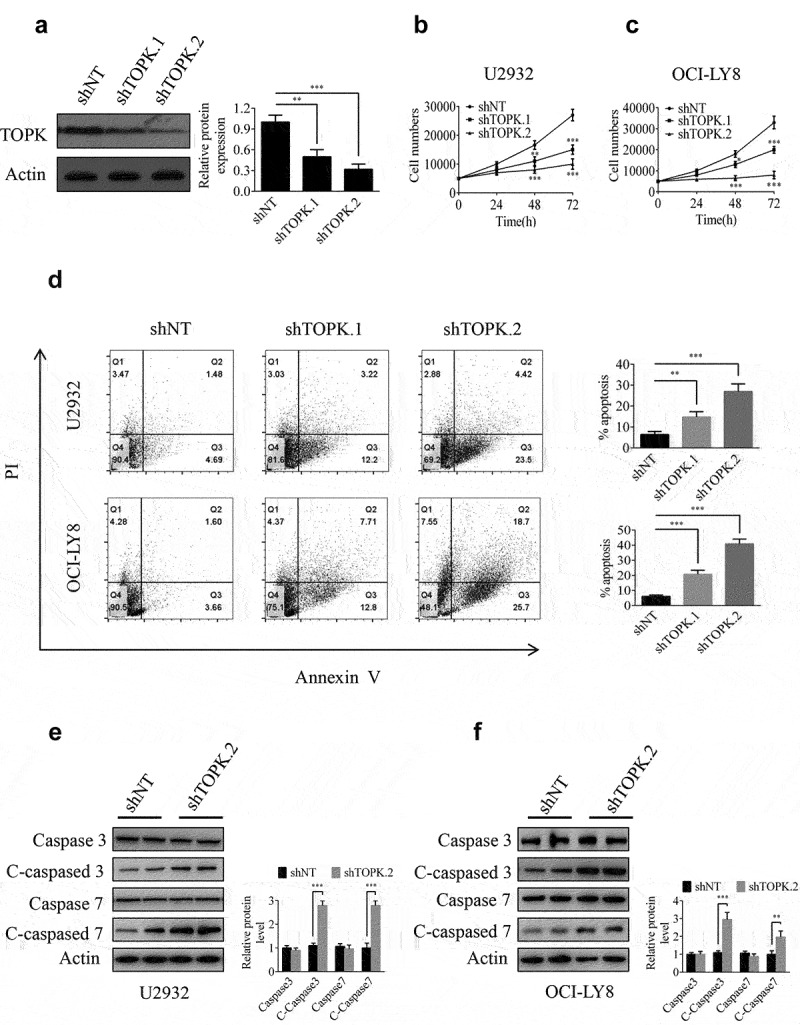
**TOPK knockdown inhibits cell growth and induces cell apoptosis in U2932 and OCI-LY8 cell lines**. (a) OCI-LY8 cells were infected with shTOPK.1, shTOPK.2 or shNT, and the TOPK knockdown efficiency was detected by Western blots. The data represent the mean ± S.D. for three individual experiments (**p < 0.01, ***p < 0.001, n = 3). (b and c) U2932 and OCI-LY8 cells were infected with shTOPK.1, shTOPK.2 or shNT, and cell proliferation was detected at 24, 48, and 72 h with the MTS assay. The data represent the mean ± S.D. for three individual experiments (*p < 0.05, **p < 0.01, ***p < 0.001, n = 3). (d) U2932 and OCI-LY8 cells were infected with shTOPK.1, shTOPK.2 or shNT for 72 h, and cell apoptosis was detected by staining with annexin V and PI (Q1 indicates dead cells, Q2 indicates late apoptotic cells, Q3 indicates early apoptotic cells, Q4 indicates viable cells). The data represent the mean ± S.D. for three individual experiments (**p < 0.01, ***p < 0.001, n = 3). (e and f) U2932 and OCI-LY8 cells were infected with shTOPK.2 or shNT, and the cleaved caspase 3 and caspase 7 protein levels were assessed by Western blots. The data are the mean ± S.D. for three individual experiments (***p < 0.001, n = 3).

To gain insight into the potential role of TOPK as an oncogene whose overexpression was associated with DLBCL tumorigenesis, we generated the TOPK knockdown cells infected with lentivirus, and the knockdown efficiency was verified in OCI-LY8 cells by Western blots (Figure 2(a)). Cell proliferation assays were performed by MTS to evaluate the effect of TOPK knockdown. The results showed that TOPK knockdown inhibited proliferation in U2932, OCI-LY8 and SUDHL-6 cells (Figure 2(b–c), Fig. S1a). As the inhibition of cell proliferation in SUDHL-6 was coincident with that in U2932 and OCI-LY8 cells, we hypothesized that the effect of TOPK would also be similar in DLBCL cell lines. Thus we chose U2932 and OCI-LY8 cells for the further study.

Studies have shown that TOPK knockdown can induce cell apoptosis [25,26]. Here, the annexin V-FITC/PI double-staining assay results showed that TOPK knockdown increased apoptosis in U2932 and OCI-LY8 cells (Figure 2(d)). We also observed that the cleavage of caspase-3 and caspase-7 was obviously enhanced in U2932 and OCI-LY8 cells by Western blots (Figure 2(e–f)). In addition, we assessed the effect of TOPK on the proliferation and apoptosis of WIL2S cells. The results showed that TOPK knockdown inhibited proliferation and induced cell apoptosis, but the effect was not as sensitive as that in U2932 and OCI-LY8 cells (Fig. S1b-1c). Overall, our data show that TOPK could modulate cell proliferation and apoptosis in DLBCL and that malignant B cells are more dependent on the TOPK pathway.

### Acetylshikonin suppresses DLBCL cell growth by targeting TOPK signaling

**Figure 3.**
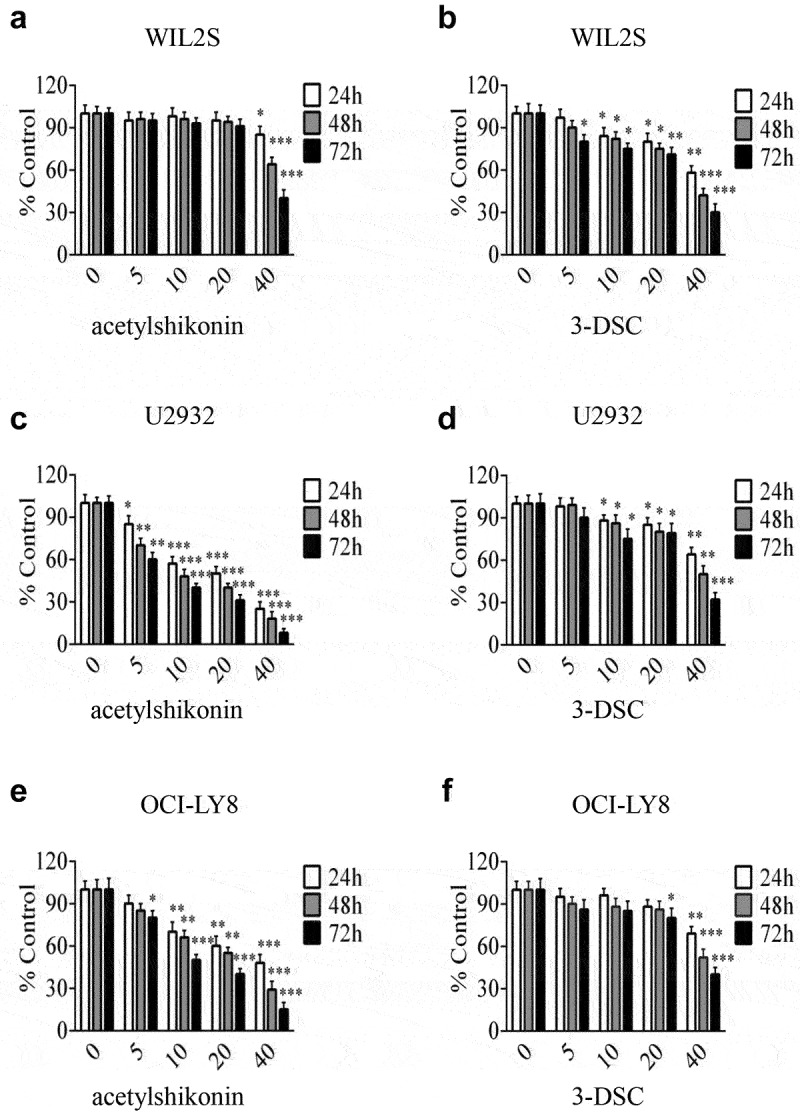
**TOPK inhibitors suppress cell proliferation in DLBCL cells**. (a-b) WIL2S cells were treated with different concentrations (5 µM, 10 µM, 20 µM, 40 µM) of acetylshikonin or 3-DSC, and cell proliferation was assessed at 24, 48, and 72 h by the MTS assay. The data represent the mean ± S.D. for three individual experiments (*p < 0.05, **p < 0.01, n = 3). (c-f) U2932 and OCI-LY8 cells were treated with different concentrations (5 µM, 10 µM, 20 µM, 40 µM) of acetylshikonin or 3-DSC, and cell proliferation was assessed at 24, 48, and 72 h by the MTS assay. The data are the mean ± S.D. for three individual experiments (*p < 0.05, **p < 0.01, ***p < 0.001, n = 3).

**Figure 4.**
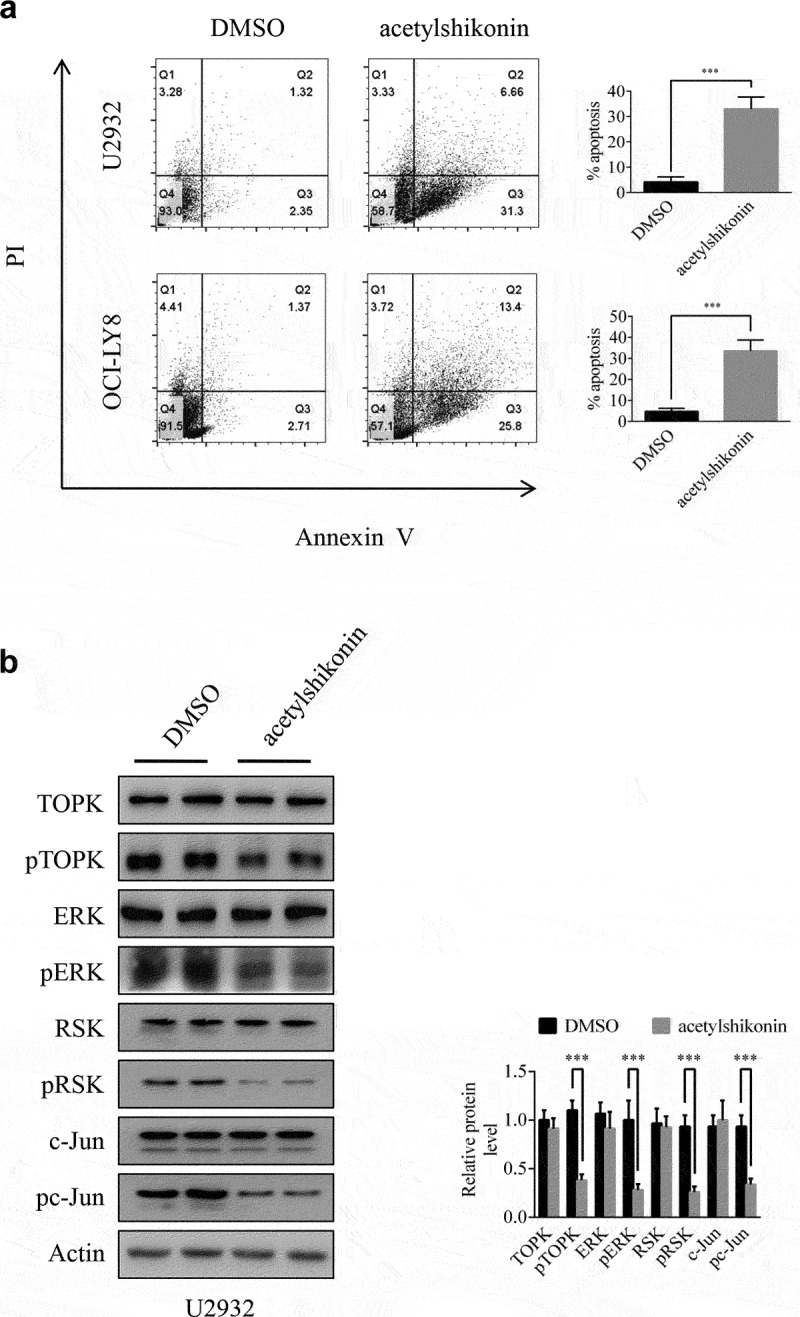
**Acetylshikonin induces DLBCL cell apoptosis and reduces the expression of proteins involved in TOPK signaling**. (a) U2932 and OCI-LY8 cells were treated with 10 µM acetylshikonin for 24 h, and cell apoptosis was detected by staining with annexin V and PI (Q1 indicates dead cells, Q2 indicates late apoptotic cells, Q3 indicates early apoptotic cells, Q4 indicates viable cells). The data represent the mean ± S.D. for three individual experiments (***p < 0.001, n = 3). (b) U2932 cells were treated with 10 µM acetylshikonin for 24 h, and the protein levels of pTOPK, total TOPK, pERK, total ERK, pRSK, total RSK, pc-Jun, total c-Jun were detected by Western blots. The data represent the mean ± S.D. for three individual experiments (***p < 0.001, n = 3).

Previous studies have shown that acetylshikonin and 3-DSC can suppress colon cancer cell growth [13,14]. To study the effects of acetylshikonin and 3-DSC on DLBCL cell growth, we assessed the effect of acetylshikonin and 3-DSC on the proliferation of WIL2S cells and PBMCs. Incubation of WIL2S cells and PBMCs with acetylshikonin (0, 5, 10, 20, 40 μM) for 24, 48 or 72 h did not show cytotoxicity up to 20 μM at 72 h (Figure 3(a), Fig. S2a); crucially, acetylshikonin inhibited the growth of DLBCL cell lines (U2932 and OCI-LY8) in a time- and dose-dependent manner (Figure 3(c–e)). However, 3-DSC showed cytotoxicity in WIL2S and U2932 cells at 10 μM for 24 h (Figure 3(b–d)), but did not show cytotoxicity in OCI-LY8 cells at 10 μM for 24 h (Figure 3(f)); thus, we estimated that DLBCL cells were more sensitive to acetylshikonin. Next, the role of acetylshikonin in cell apoptosis was examined. Treatment with 10 μM acetylshikonin at 24 h induced cell apoptosis in U2932 and OCI-LY8 cells (Figure 4(a)). In addition, we investigated whether acetylshikonin affected the TOPK signaling pathway, and we determined the effects of acetylshikonin on the expression of phosphorylated TOPK (pTOPK), phosphorylated ERK (pERK), phosphorylated ribosomal S-6 kinase (pRSK) and phosphorylated c-Jun (pc-Jun). Treatment of U2932 and OCI-LY8 cells with 10 μM acetylshikonin at 24 h decreased the levels of pTOPK, pERK, pRSK, pc-Jun, the total levels of TOPK, ERK, RSK and c-Jun were unchanged compared to those in the DMSO controls (Figure 4(b), Fig. S3a).

**Figure 5.**
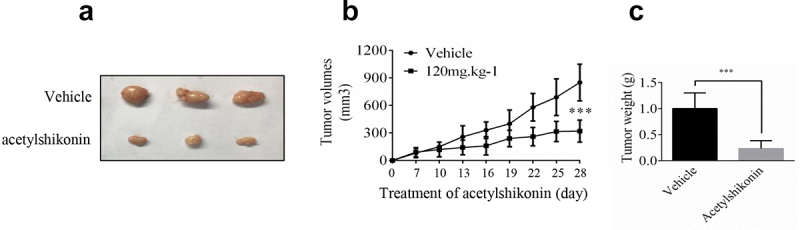
**Acetylshikonin inhibits DLBCL cell growth *in vivo***. (a-b) The effect of acetylshikonin on the size of tumors was assessed. Vehicle or acetylshikonin (120 mg.kg^−1^ body weight) was administered by gavage. Data are shown as mean values ± SD, from n = 6 in each group. (c) Tumor weight was measured after treatment on the last day of the study. Data are shown as mean values ± SD (***p < 0.001, n = 6).

In order to verify our findings, we used nude mice to examine the anti-tumor activity of acetylshikonin *in vivo*, acetylshikonin observably inhibited U2932 cells tumorigenicity in nude mice (Figure 5(a–c)).

### The effect of acetylshikonin is dependent on the expression of TOPK

**Figure 6.**
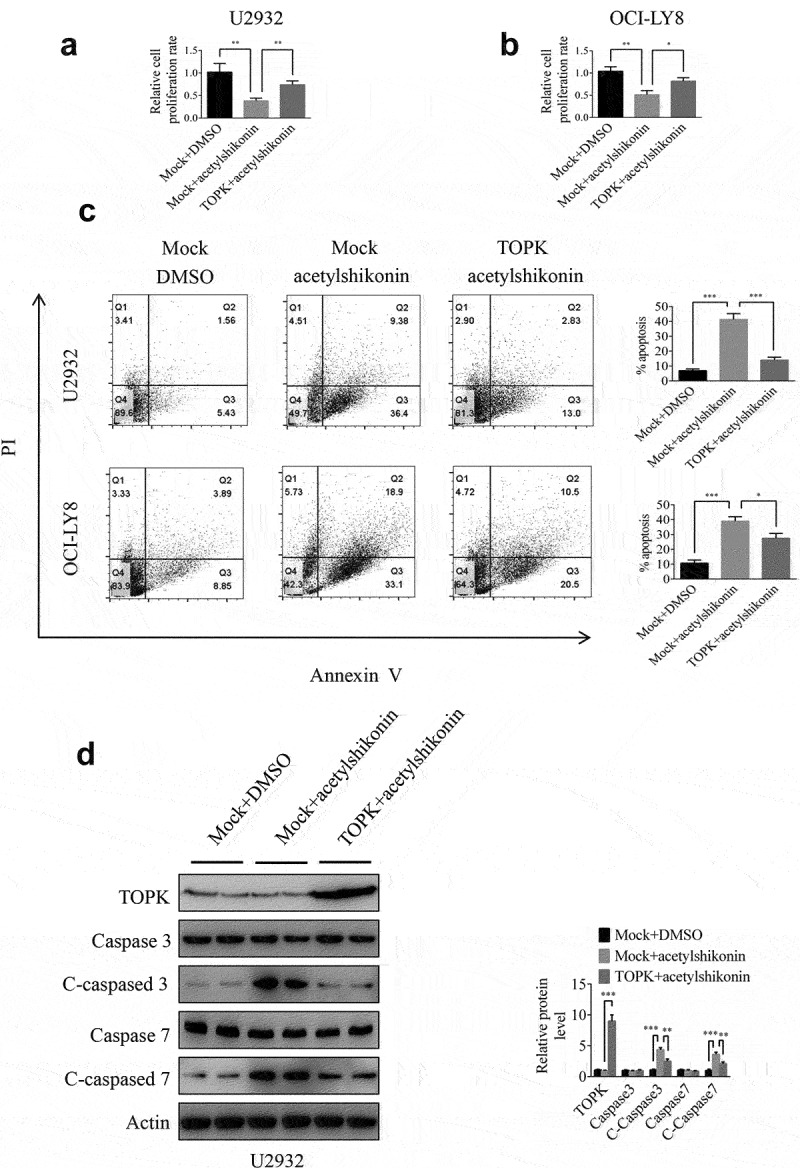
**TOPK overexpression attenuates the effect of acetylshikonin**. (a-b) U2932 and OCI-LY8 cells were infected with a lentivirus carrying TOPK cDNA or treated with 10 µM acetylshikonin, and cell growth was determined at 72 h using the MTS assay. The data represent the mean ± S.D. for three individual experiments (*p < 0.05, **p < 0.01, n = 3). (c) U2932 and OCI-LY8 cells were infected with a lentivirus carrying TOPK cDNA or treated with 10 µM acetylshikonin, and cell apoptosis was detected by staining with annexin V and PI (Q1 indicates dead cells, Q2 indicates late apoptotic cells, Q3 indicates early apoptotic cells, Q4 indicates viable cells). The data represent the mean ± S.D. for three individual experiments (*p < 0.05, ***p < 0.001, n = 3). (d) U2932 cells were infected with a lentivirus carrying TOPK cDNA or treated with 10 µM acetylshikonin, and the cleaved caspase 3 and caspase 7 protein levels were assessed by Western blots. The data represent the mean ± S.D. for three individual experiments (**p < 0.01, ***p < 0.001, n = 3).

To further determine whether TOPK could mediate acetylshikonin-induced cell growth inhibition, we infected TOPK cDNA in U2932 and OCI-LY8 cells and treated them with acetylshikonin, followed by analysis of cell proliferation and apoptosis. The TOPK overexpression efficiency was shown in U2932 and OCI-LY8 cells by Western blots (Figure 6(d), Fig. S4c) and the expression of pTOPK was shown in Fig. S4a-4b. The results showed that the inhibition of cell proliferation treated with acetylshikonin was partly rescued upon overexpression of TOPK (Figure 6(a–b)) and the induction of cell apoptosis treated with acetylshikonin was partly inhibited by the overexpression of TOPK (Figure 6(c)). The cleavage of caspase-3 and caspase-7 showed coincident results (Figure 6(d), Fig. S4c). In addition, the inhibition of cell proliferation treated with acetylshikonin could not be rescued by the knockdown of TOPK in U2932 and OCI-LY8 cells (Fig. S4d-4e). These data indicated that the mechanism of acetylshikonin action is highly dependent on the expression of TOPK.

## Discussion

Despite improvements in the treatments of DLBCL, it is still associated with a high mortality rate [27]; thus, exploring novel factors that affect DLBCL progression are important for targeted therapy. This study reports that TOPK has a key role in DLBCL tumorigenic properties. Acetylshikonin suppresses DLBCL cell growth by targeting the TOPK signaling pathway and may be a potential treatment strategy for DLBCL.

TOPK is an active form of MEK1 that can phosphorylate ERK1/2^12^. Studies have shown the correlation between TOPK expression and poor prognosis in several cancers, such as breast cancer [28], lung cancer [29], leukemia [30], and burkitt’s lymphoma [9]. The overexpression of TOPK is associated with the tumorigenesis [31]. Although TOPK plays important roles in the development of in multiple cancers, its function in DLBCL remains unclear. This work evaluates the function of TOPK in DLBCL.

Our results indicated that TOPK was highly expressed in DLBCL, both in DLBCL cell lines and tissues (Figure 1). For the functional study, we demonstrated that TOPK knockdown dramatically inhibited cell proliferation and induced cell apoptosis in U2932 and OCI-LY8 cells (Figure 2, Fig. S1a). However, the function was not sensitive in WIL2S cells (Fig. S1b-1c), which indicated that malignant B cells were more dependent on the TOPK pathway. These results suggest that TOPK knockdown obviously attenuates the malignant phenotypes of DLBCL cells, and thus, searching for appropriate inhibitors that could target TOPK is pregnant for DLBCL treatment.

An ideal anticancer compound should lack cytotoxicity against normal cells. In this study, we chose two native compounds (acetylshikonin and 3-DSC) that were extracted from plants and showed anticancer effects by targeting TOPK [13,14]. Our data indicated that acetylshikonin showed no cytotoxicity up to 20 μM at 72 h in WIL2S cells and PBMCs (Figure 3(a), Fig. S2a), but 3-DSC showed cytotoxicity in WIL2S cells up to 10 μM at 24 h (Figure 3(b)). After incubation with acetylshikonin or 3-DSC (0, 5, 10, 20, 40 μM) for 24, 48 or 72 h, acetylshikonin showed greater inhibition of cell proliferation than 3-DSC in U2932 and OCI-LY8 cells (Figure 3(c–f)); thus, we estimated that acetylshikonin was a better targeted treatment for DLBCL. The observed anticancer effects of acetylshikonin were reported in human pancreatic Panc-1 cancer cells, colon cancer cells, leukemia and hepatocellular carcinoma [13,32–34]. In our studies, we demonstrated that acetylshikonin inhibited cell proliferation (Figure 3(c–e)) and induced cell apoptosis (Figure 4(a)) in U2932 and OCI-LY8 cells. Studies have shown that TOPK is a major target of acetylshikonin that mediates the inhibition of proliferation and induction of apoptosis, several signaling molecules including ERK, ribosomal S-6 kinase (RSK), c-Jun and transcription factor (NF-κB) are involved in TOPK signaling and acetylshikonin decreases the phosphorylation of TOPK, ERK, RSK, c-Jun and the transcriptional activity of NF-κB [13]. Our results confirmed that acetylshikonin could decrease the expression of pTOPK, pERK, pRSK and pc-Jun in U2932 and OCI-LY8 cells (Figure 4(b), Fig. S3a). We also used nude mice to examine the anti-tumor activity of acetylshikonin *in vivo*, our results showed that acetylshikonin observably inhibited U2932 cells tumorigenicity in nude mice (Figure 5).

In addition, TOPK overexpression abolished inhibition of the cell proliferation, induction of cell apoptosis and the increase in cleaved caspase-3 and caspase-7 expression induced by treatment with acetylshikonin (Figure 6, Fig. S4c), but the inhibition of cell proliferation after treatment with acetylshikonin could not be rescued by the knockdown of TOPK in U2932 and OCI-LY8 cells (Fig. S4d-4e), which further confirmed that TOPK is involved in the acetylshikonin induced cell growth inhibition.

## Conclusion

The present study suggests that TOPK may mediate acetylshikonin induced cell growth inhibition in DLBCL. Our results demonstrated that TOPK expression was upregulated in DLBCL and involved in the tumorigenicity of DLBCL, which might provide a novel potential target for DLBCL therapy. As acetylshikonin inhibits cell proliferation and induces cell apoptosis in DLBCL, this compound could be considered a viable therapeutic option for the treatment of DLBCL and as a DLBCL therapeutic in further clinical research.

## Supplementary Material

Supplemental Material

## Highlights

1. TOPK is expressed at high levels in DLBCL.

2. Silencing TOPK inhibits cell growth and induces cell apoptosis of DLBCL.

3. Acetylshikonin suppresses DLBCL cell growth by targeting TOPK signaling and the effect of acetylshikonin is dependent on the expression of TOPK.
