# Supplementary figures and images for: Acetylshikonin suppresses diffuse large B-Cell Lymphoma cell growth by targeting the T-lymphokine-activated killer cell-originated protein kinase signalling pathway
Source: Bioengineered. 2022 Feb 9;13(2):4428–40. doi: 10.1080/21655979.2022.2034584 (PMC8973784; doi:10.1080/21655979.2022.2034584)

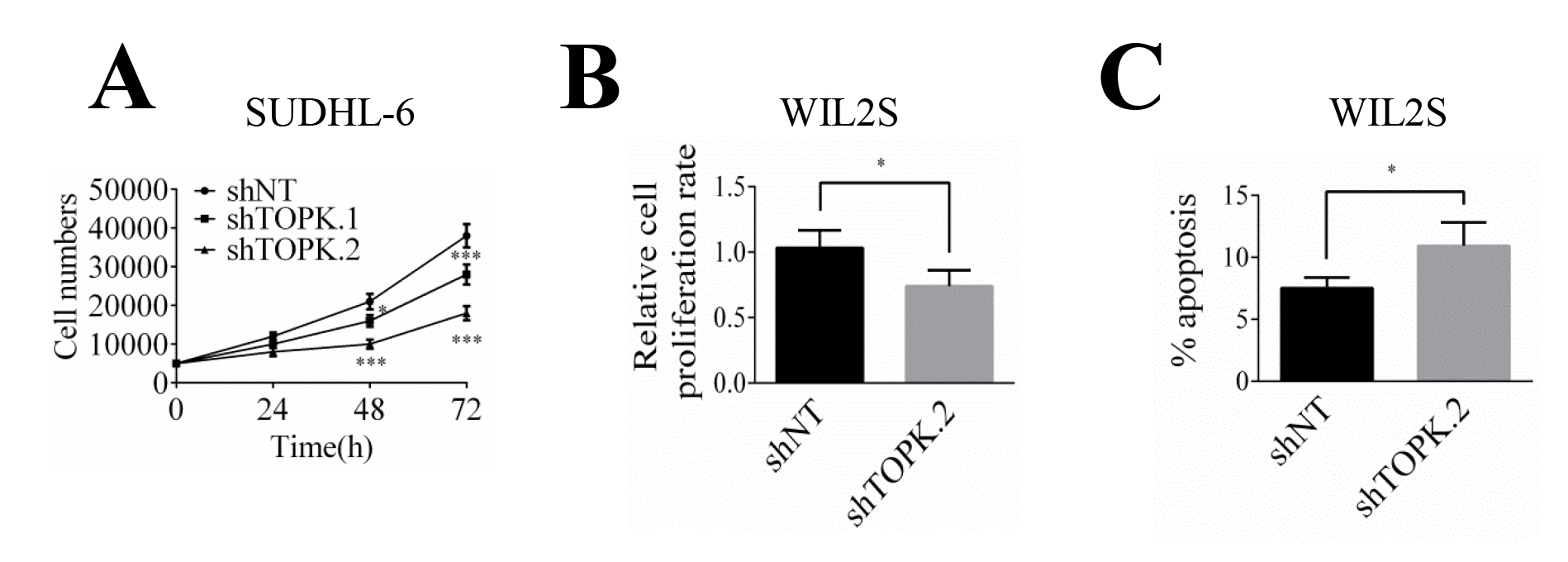

Supplement: Supplemental Material [file KBIE_A_2034584_SM6811.zip › supplementary/Figure S1.jpg]

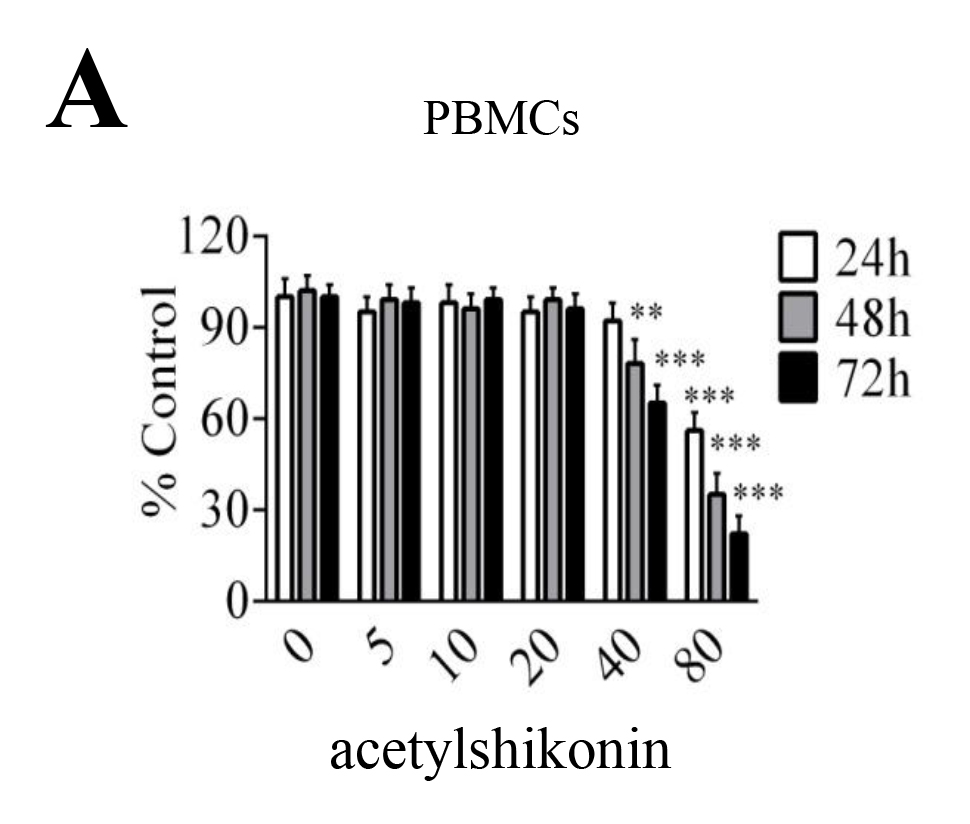

Supplement: Supplemental Material [file KBIE_A_2034584_SM6811.zip › supplementary/Figure S2.jpg]

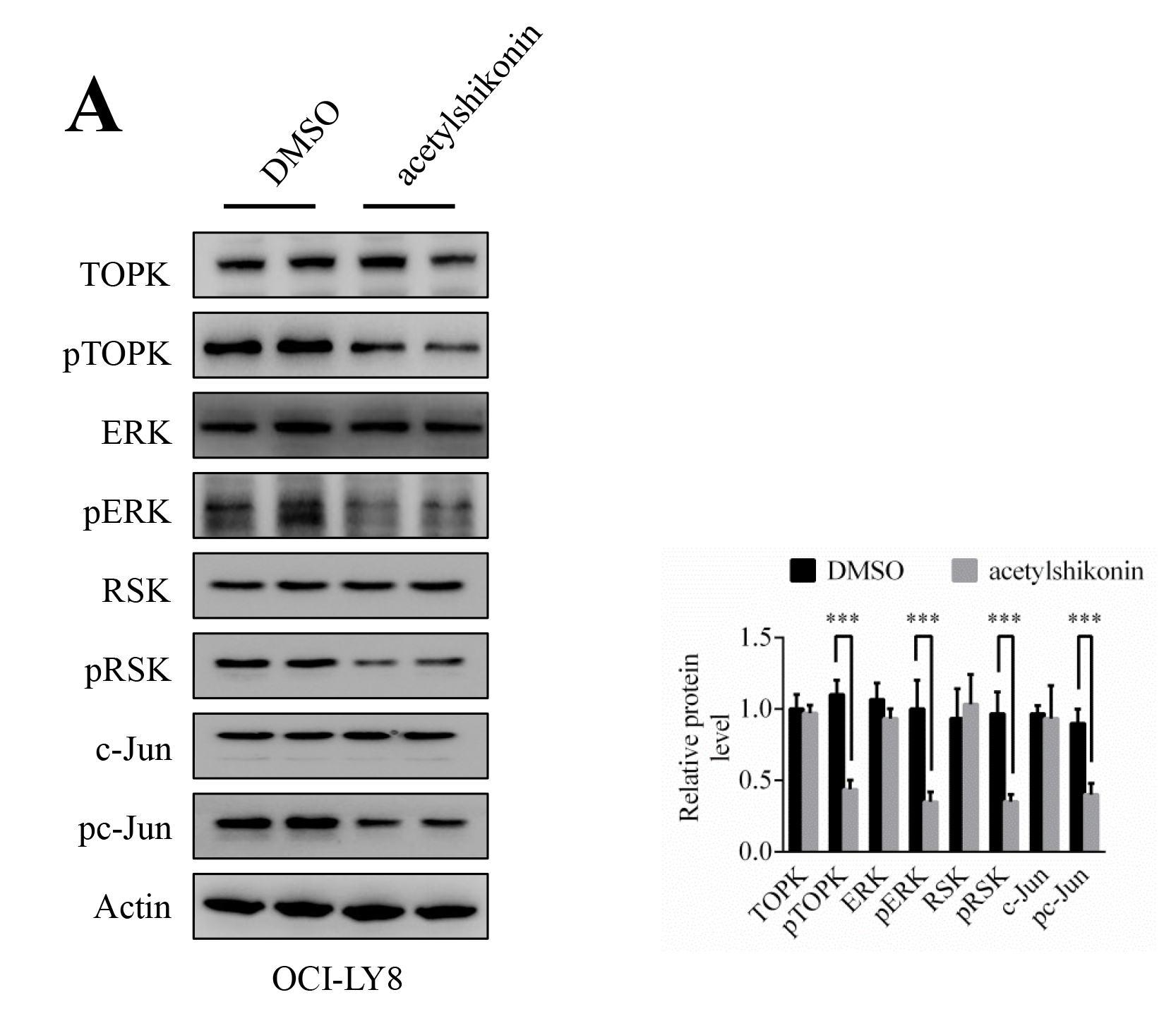

Supplement: Supplemental Material [file KBIE_A_2034584_SM6811.zip › supplementary/Figure S3.jpg]

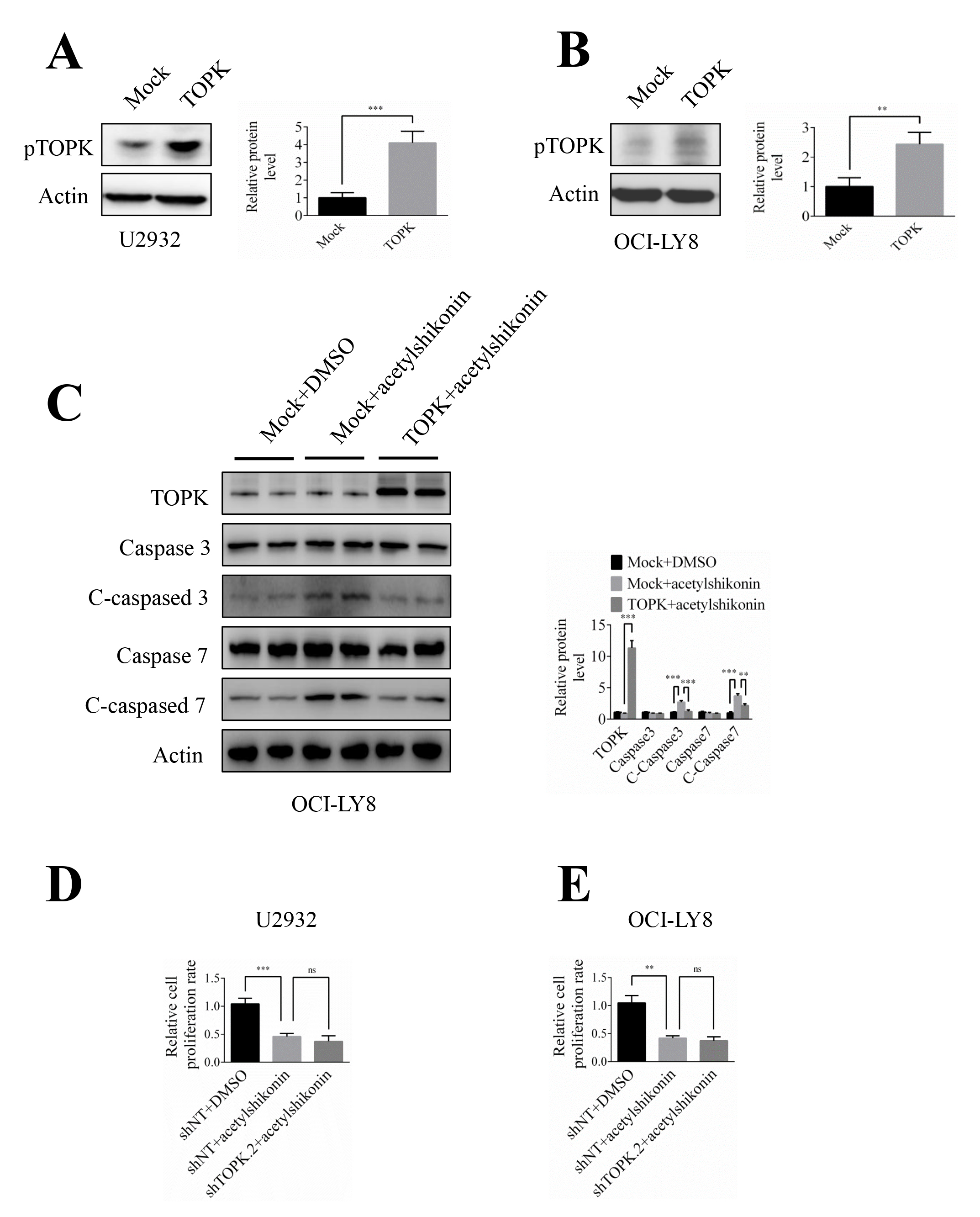

Supplement: Supplemental Material [file KBIE_A_2034584_SM6811.zip › supplementary/Figure S4.jpg]
